# Postoperative Vision-Related Quality of Life After Sphenoid Wing Meningioma Surgery: Impact of Radiomic Shape Features and Age

**DOI:** 10.3390/jcm14010040

**Published:** 2024-12-25

**Authors:** Alim Emre Basaran, Martin Vychopen, Clemens Seidel, Alonso Barrantes-Freer, Felix Arlt, Erdem Güresir, Johannes Wach

**Affiliations:** 1 Department of Neurosurgery, University Hospital Leipzig, 04103 Leipzig, Germany; 2 Comprehensive Cancer Center Central Germany, Partner Site Leipzig, 04103 Leipzig, Germanyalonso.barrantes-freer@medizin.uni-leipzig.de (A.B.-F.); 3 Department of Radiation Oncology, University Hospital Leipzig, 04103 Leipzig, Germany; 4 Department of Neuropathology, University Hospital Leipzig, 04103 Leipzig, Germany

**Keywords:** meningioma, quality of life, visual dysfunction, radiomics

## Abstract

**Background:** Sphenoid wing meningiomas (SWM) frequently compress structures of the optic pathway, resulting in significant visual dysfunction characterized by vision loss and visual field deficits, which profoundly impact patients’ quality of life (QoL), daily activities, and independence. The objective of this study was to assess the impact of SWM surgery on patient-reported outcome measures (PROMs) regarding postoperative visual function. **Methods:** The Visual Function Score Questionnaire (VFQ-25) is a validated tool designed to assess the impact of visual impairment on quality of life. The questionnaire was distributed to a previously published study population in which shape radiomics were correlated with new cranial nerve deficits after SWM surgery. **Results:** A total of 42 patients (42/74; 56.8%) responded to the questionnaire. Of the 42 patients, 30 were female (71%) and 12 were male (29%). The multivariable analysis demonstrated that lower sphericity reflecting irregular SWM shape was associated with poorer VFQ-25 (OR: 6.8, 95% CI: 1.141.8, *p* = 0.039), while age was associated with lower VFQ-25 (OR: 27, 95% CI: 2.7−272.93, *p* = 0.005), too. Analysis of the subcategories of the VFQ-25 revealed significantly reduced general vision (*p* = 0.045), social functioning (*p* = 0.045), and peripheral vision (*p* = 0.017) in those with SWM with low sphericity. **Conclusions:** The study highlights that SWM surgery impacts postoperative visual function, with age and irregular SWM shape being associated with poorer postoperative VFQ-25 scores. VFQ-25 is a feasible tool to assess vision outcome in SWM surgery and has clinical potential for longitudinal follow-up evaluations. Irregular SWM shape should be considered during preoperative treatment planning and patient consultation regarding functional outcome.

## 1. Introduction

Sphenoid wing meningiomas (SWM) account for about 15–25% of meningiomas and are classified into three categories based on their anatomical structure: lateral, middle, and median [1]. A comprehensive study analyzing 256 sphenoid wing meningiomas revealed that 42% (108 cases) were medial sphenoid wing meningiomas (MSWM) [2]. In most cases, complete surgical removal with treatment of the dural attachment in meningioma is the therapy of choice, guided by the Simpson grading system. The Simpson grading system classifies the Extent of Tumor Resection (EoR) into five grades [3]. Recent high-class data from 1686 tumors showed that dural margin treatment (Simpson grade I/II) prolonged progression-free survival and is still of paramount importance even in the molecular era of meningiomas [4]. However, given the complex associated anatomy and potential tumor adherence to critical structures, performing Simpson grade I resection with an increased risk of new cranial nerve deficits in MSWM is not always recommended [5].

Long-term harm is possible despite MSWM’s slow growth because of the potential compression of relevant adjacent neurovascular structures like the cavernous sinus and optic nerve [6]. Visual impairment is among the most frequent symptomatic burdens of MSWM.

The quality of life (QoL) of MSWM patients is greatly impacted by deterioration in visual function. The ability to perform daily activities can be significantly hampered by these visual impairments [1,7,8]. In their work, Chaichana et al. presented the results of their study, in which 81 patients with a MSWM were examined. The study showed that 55% of patients experienced a decrease in visual acuity [9]. Assessment of postoperative vision for pituitary adenomas has already been established using the Visual Function Questionnaire 25 (VFQ-25) [10].

The amount of research investigating the connection between tumor shape and postoperative neurological outcomes has significantly increased in the last few years [11,12]. An irregular tumor shape is typically characterized by: (1) non-spherical or non-oval appearance, (2) presence of structural indentations and protrusions along the tumor border, and (3) lack of circumscribed (well-defined) margins [12,13]. The shape of MSWM is further characterized using sphericity, a metric that quantifies how closely an object’s shape approximates a perfect sphere. Sphericity is determined by comparing the surface area of a sphere with the same volume as the object to the actual surface area of the object. For instance, the sphericity of a meningioma is calculated by evaluating the ratio of the surface area of a sphere with the tumor’s volume to the tumor’s surface area. The calculation follows the Hakon Wadell method, using the formula: sphericity = π1/3 (6 × tumor volume)2/3/surface area. A sphericity value of 1 indicates a perfect sphere, while lower values reflect increasingly irregular shapes [14]. In a previous institutional study, it was demonstrated that irregular shape in MSWMs is independently associated with higher postoperative cranial nerve deficits, especially in the oculomotor nerve, along with increased proliferative activity and shorter progression-free survival [13].

Visual evoked potentials (VEPs) represent a valuable intraoperative real-time warning tool regarding visual impairment in skull base surgery [15]. VEPs may prove particularly beneficial in the detection of subclinical visual dysfunction and the monitoring of postoperative recovery, particularly in cases where patients may experience difficulty in cooperating with subjective visual tests [16].

Although data on the QoL of MSWM patients without visual impairment are limited, studies on meningioma patients suggest reduced QoL due to neurological symptoms and the psychological impact of diagnosis [17]. Fisher et al. found significant differences in HRQOL between posterior and anterior/middle skull base meningiomas, with worse outcomes linked to radiotherapy [18]. Postoperative visual deterioration occurred in 22–35% of MSWM surgery patients, while 70–71% maintained normal visual acuity, and 8–10% experienced an improvement [9,19].

Previous studies have primarily focused on overall QoL in general [8,17] and not on the specific functional deterioration associated with MSWM. To the best of our knowledge, no prior study has examined the vision-related (VR)-QoL in patients with MSWM using internationally validated and accepted questionnaires. The aim of the study was to evaluate the VR-QoL of patients with MSWM and to determine the factors affecting postoperative outcomes.

## 2. Materials and Methods

### 2.1. Study Design and Patient Selection

**Figure 1:**
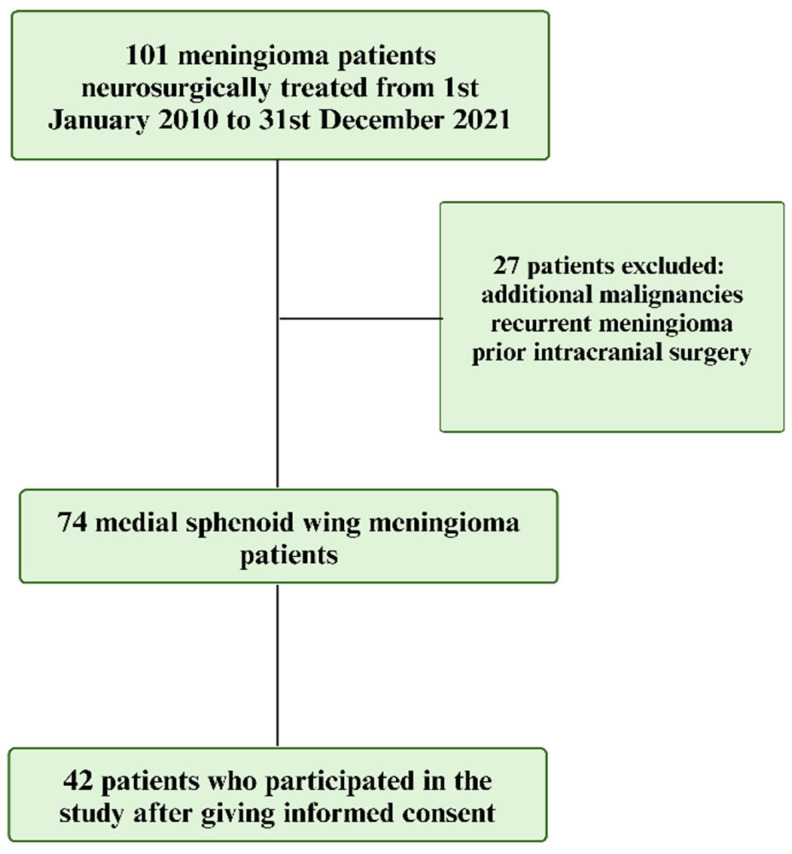
Flowchart summarizing the selection process of participants for the study on MSWM. Out of 101 neurosurgically treated patients from January 2010 to December 2021, 27 were excluded due to additional malignancies, recurrent meningioma, or prior intracranial surgery. The final cohort included 74 patients with MSWM, of which 42 provided informed consent to participate in the study.

The present study used retrospective QoL data without preoperative assessment. All patients who underwent MSWM surgery between 2010 and 2021 were selected from the institution’s consecutive meningioma database, which included those neuropathologically diagnosed with meningioma and who underwent surgery for MSWM. After exclusions, a total of 74 patients met the inclusion criteria for the study. Subsequently, the patients were contacted by telephone and informed about the study. After informed consent and the completion of the voluntary participation process, 42 patients remained. A flowchart about the patient selection process is demonstrated in Figure 1. The study cohort comprised those from a previous publication. Ethical approval for the study was obtained from the local Institutional Review Board (IRB) under approval number 165/24-ck. The ethical approval date was 16 May 2024.

### 2.2. Inclusion and Exclusion Criteria

The patients included in the study were diagnosed with MSWM according to neuroradiological criteria and classified as meningiomas based on the 2016 World Health Organization (WHO) classification [20]. Inclusion criteria required patients to be aged between 18 and 70 at the time of surgery. Patients were excluded from the study if the precise neuroanatomical localization of MSWM could not be determined, if they declined to participate, or if they did not complete and return the questionnaire in its entirety. Additionally, patients with other malignancies, recurrent meningiomas, or a history of prior intracranial surgery were also excluded.

### 2.3. MRI Evaluation

The location of the meningioma, the presence of peritumoral edema, tumor volume, and surface area were assessed using pre- and postoperative MRI scans. These scans were analyzed using Gd-enhanced T1-weighted sequences with 3D Slicer software (Version 5.2.1, Surgical Planning Laboratory, Harvard University, Cambridge, MA, USA). The detailed workflow of segmentation and calculation of sphericity was described previously [13]. Sphericity was determined using the Hakon Wadell method, and calculated with the following formula: Sphericity = π1/3 (6 × tumor volume)2/3/surface area. A sphericity value of 1 indicated a perfect sphere, while lower values reflected increasingly irregular shapes [14].

### 2.4. Tumor Resection Classification

The extent of tumor resection was classified according to the Simpson classification system [3].

### 2.5. Questionnaire

The online questionnaire, based on the VFQ-25 questions, was created using the Google Forms platform accessed on 16 May 2024: https://docs.google.com/forms/d/1InoVa4AN-jMwjxasqMXcKNvIRx7Cg9GejRj-UTKetbo/prefill. The questionnaire used the validated VFQ-25 score, which has been used in numerous clinical studies [21,22,23]. The VFQ-25 measures both vision-specific functioning and overall well-being through responses to 25 items, which are grouped into specific subscales. For German-speaking patients, a validated German translation ensured reliability and interpretability. The translation was performed using DeepL Translator. The validity and reliability of DeepL translator for medical translation has previously been proven [24]. After patients who met the inclusion criteria were selected from the meningioma database, they were contacted by telephone. At the beginning of the interview, it was emphasized that the conversation and answers would remain anonymous, and that participation was completely voluntary. The aims and purpose of the study were explained, and after patients provided verbal consent to participate, the questionnaire was sent to them via email.

### 2.6. Statistics

All statistical analyses were performed using SPSS version 29.0 (IBM, Armonk, NY, USA). The statistical analysis of the VFQ-25 scores was performed in accordance with the scoring system delineated in the NEI-VFQ-25 manual [25]. The VFQ-25 consists of 25 core items organized into 12 subscales that assess the following domains: general health, general vision, ocular pain, near activities, distance activities, social functioning, mental health, role difficulties, dependency, driving, color vision, and peripheral vision. Each item is scored using a Likert scale, with response options ranging from a high degree of difficulty to no difficulty or no impact on visual function. The responses for individual items, as raw scores, were transformed onto a scale from 0 to 100, where 100 represented the optimal level of function, and 0 represented the worst possible function. To calculate the subscale scores, each individual item within a subscale was averaged to produce a subscale score. The subscale scores were then transformed to a scale ranging from 0 to 100. To represent overall visual functioning, a composite score was calculated as the average of the 11 vision-specific subscale scores, excluding the general health subscale. This composite score, ranging from 0 to 100, provided an overall measure of the patient’s VR-QoL. Higher subscale scores indicated better performance or less impairment in the respective domain. The composite score reflected the patient’s overall visual function and postoperative QoL, with higher scores denoting better visual function. Subsequently, the total composite score was dichotomized. The analyses were evaluated using the *t*-test or Mann–Whitney U-test, depending on their distribution. The predictive accuracy of age and sphericity for postoperative visual outcomes was evaluated using receiver operating characteristic (ROC) curves, and the area under the curve (AUC) with 95% confidence intervals was calculated. Youden’s index was also defined as a summary measure of the effectiveness of a diagnostic test, defined as J = Sensitivity + Specificity − 1, and it identified the optimal cut-off point that maximizes the discriminative ability in ROC curve analysis [26]. Multivariate logistic regression was employed to identify independent predictors of poor visual function outcomes. Variables included in the multivariate analysis were those found to be statistically significant in univariate analysis. Sex and cavernous sinus invasion were also included in multivariable analysis to adjust the results for a female predominance among meningioma patients and the anatomical course of vision-related cranial nerves in the cavernous sinus. Odds ratios (OR) were presented with 95% confidence intervals. A *p*-value of less than 0.05 was considered statistically significant for all tests. ROC curves and bar graphs were created using SPSS, while the forest plot was generated using the R package *ggplot2* (R Foundation for Statistical Computing, Vienna, Austria).

## 3. Results

### 3.1. Patient Characteristics

The study included a total of 42 MSWM patients participating in the questionnaire. The mean age of the patients was 57.9 ± 12.9 years. Of the 42 patients, 30 were female (71.4%) and 12 were male (28.6%). According to the 2016 WHO classification system, 39 patients (92.9%) were diagnosed with grade 1 meningioma, while three patients (7.1%) were diagnosed with grade 2 meningioma. The mean tumor volume was 23.2 cm^3^ (standard deviation (SD) ± 27.9).

Based on the Simpson resection classification, no patient underwent a Simpson grade I resection, 28 patients (66.6%) underwent grade II resection, two (4.8%) had grade III resection, and 12 (28.6%) received a grade IV resection. Neuroradiological examination revealed peritumoral edema in 13 patients (31%). Cavernous sinus infiltration was observed in seven patients (16.7%). Two patients (4.8%) received adjuvant radiotherapy postoperatively. Regarding laterality, 20 patients (47.6%) had right-sided and 22 patients (52.4%) left-sided MSWM. The median time from surgery to interview was 108 months (SD ± 46.9). A summary of additional patient characteristics can be found in Table 1.

### 3.2. Univariate Analysis of Visual Function

**Figure 2:**
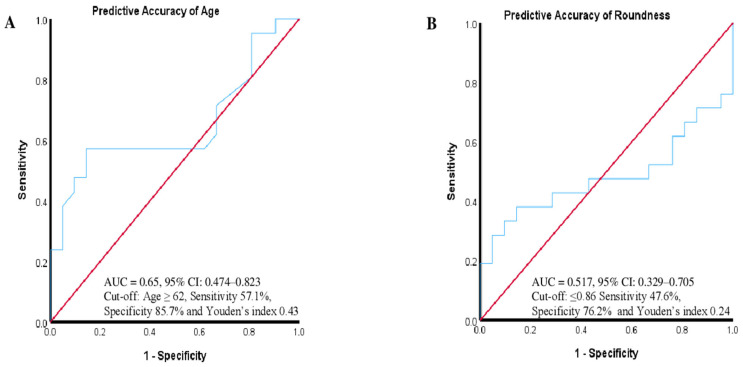
(**A**) The ROC curve shows the discriminative ability of patient age at the time of surgery to predict postoperative visual outcomes. The area under the curve (AUC) is 0.65, with a 95% Confidence Interval (CI) ranging from 0.474 to 0.823, indicating moderate predictive accuracy. A cut-off value of age ≥ 62 years was determined, yielding a sensitivity of 57.1% and a specificity of 85.7%. The Youden’s index, which balances sensitivity and specificity, is 0.43. (**B**) The ROC curve evaluates the ability of tumor sphericity to predict postoperative visual outcomes. The AUC for tumor sphericity is 0.52, with a 95% CI ranging from 0.329 to 0.705, indicating low predictive accuracy. A cut-off value of sphericity ≤ 0.86 was identified, resulting in a sensitivity of 47.6% and a specificity of 76.2%. The Youden’s index for this model is 0.24. Both panels display ROC curves (light blue) plotted against a reference line (red) representing random chance, with the x-axis showing 1-specificity and the y-axis showing sensitivity.

Regarding patient-reported VR-QoL, 21 patients (50%) showed improvement postoperatively, while 21 patients (50%) experienced a subjective deterioration of vision-related QoL. A ROC curve was created for age and tumor sphericity. Predictive accuracy for age was assessed using the AUC-ROC, which yielded a value of 0.65 (95% CI: 0.474–0.823) (Figure 2A). In comparison, prediction based on tumor sphericity showed a lower AUC-ROC of 0.52 (95% CI: 0.329–0.705) (Figure 2B). The cut-off for age was ≥62, which resulted in a sensitivity of 57.1%, specificity of 85.7%, and a Youden’s index of 0.43 (Figure 2A). For tumor sphericity, a cut-off value of ≤0.86 was used, which showed a sensitivity of 47.6%, specificity of 76.2%, and a Youden’s index of 0.24 (Figure 2B).

Those patients with a lower sphericity (≤0.86) had a mean total VFQ-25 score of 73.4 ± 23.3, whereas more regularly shaped MSWMs with a sphericity > 0.86 had a mean VFQ-25 score of 86.8 ± 17.8 (*p* = 0.04). Patients < 62 years had a mean total VFQ-25 score of 88.0 ± 15.4, and those aged ≥ 62 years at surgery had a mean total VFQ-25 score of 71.2 ± 25.0, respectively (*p* = 0.03). Further analyses revealed no statistically significant correlation between axial diameter (*p* = 0.69), peritumoral edema (*p* = 0.329), tumor calcification (*p* = 0.323), adjuvant radiotherapy (*p* = 1.000), the side of the MSWM (*p* = 0.806), or cavernous sinus infiltration (*p* = 0.792) with VFQ-25 scores. Furthermore, an analysis of the subcategories of the VFQ-25 showed a significant reduction in general vision (*p* = 0.014), social functioning (*p* = 0.045), and peripheral vision (*p* = 0.017) postoperatively in those patients with low tumor sphericity. Additionally, higher age at surgery was associated with statistically significant worsening in QoL postoperatively in general vision (*p* = 0.001), near activities (*p* = 0.006), distance activities (*p* = 0.006), mental health (*p* = 0.037), role difficulties (*p* = 0.020), dependency (*p* = 0.024), and color vision (*p* = 0.001).

**Figure 3:**
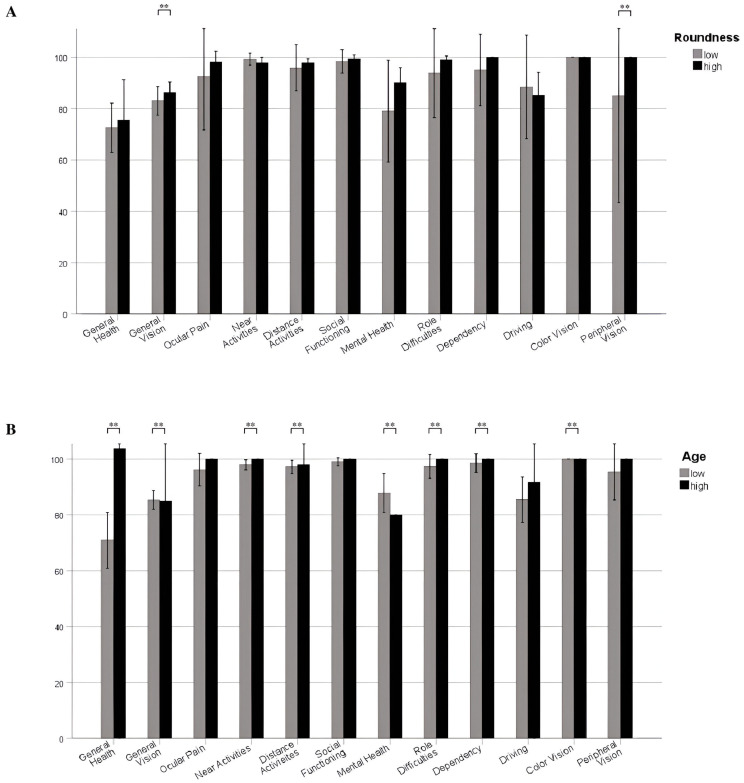
(**A**) Compares the postoperative visual function scores between patients with low and high tumor sphericity across the same health domains. Asterisks (**) above the columns indicate statistically significant differences in visual outcome scores between different daily health domains. In both figures, the height of bars represents the visual function scores for each group, and error bars show the standard deviation, illustrating variability within each group. (**B**) Compares the postoperative visual function scores between patients in low and high age groups across the same health domains.

The bar graph in Figure 3A,B illustrates the influence of age and tumor sphericity on various subcategories of the VFQ-25 assessment of QoL in patients with MSWM.

### 3.3. Multivariate Analysis

The following parameters were considered in the multivariate regression analysis: age, sex, sphericity, and cavernous sinus invasion.

**Figure 4:**
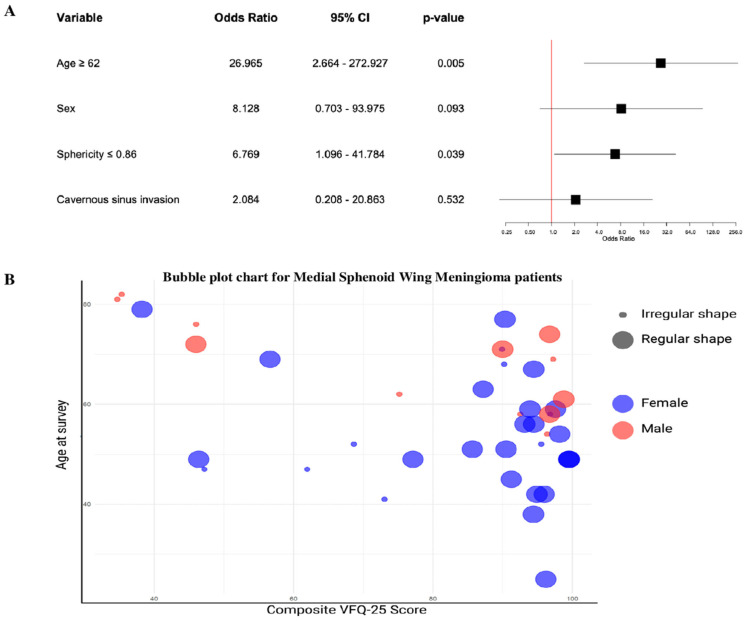
(**A**) A forest plot showing factors associated with postoperative VR-QoL in MWSM, with odds ratios (OR) and 95% confidence intervals (CI) from a multivariable logistic regression. The red dashed line at OR = 1.00 represents no effect. Significant predictors include age (cut-off ≥ 62; OR = 26.965, 95% CI: 2.664–272.927, *p* = 0.005) and sphericity (cut-off ≤ 0.86; OR = 6.769, 95% CI: 1.096–41.784, *p* = 0.039). Non-significant factors such as sex and cavernous sinus invasion had CIs crossing the null line. (**B**) A bubble plot correlating total composite VFQ-25 scores (x-axis) with age (y-axis), with colors representing sex (red: males, blue: females) and bubble size reflecting tumor shape (larger: regular, smaller: irregular). The plot highlights variations in QoL scores by tumor shape, sex, and age, suggesting interdependencies affecting VR-QoL.

The results of the multivariate logistic regression analysis show that older age (≥62 years) at surgery has a significant influence on VFQ-25 scores (OR = 26.97, 95% CI 2.67–272.93, *p* = 0.005). Furthermore, low tumor sphericity (≤0.86) is statistically significantly associated with poor VFQ-25 scores (OR = 6.77, 95% CI 1.10–41.78, *p* = 0.039). In addition, the analysis considered variables such as cavernous sinus invasion and sex, although these did not reach statistical significance (Figure 4A). Additionally, the bubble plot in Figure 3B compares Composite VFQ-25 score with age at surgery, with the data points color-coded by sex and size-coded by tumor shape: larger bubbles represents regular shape and smaller bubbles represents irregular shape. Furthermore, data points also represent sex, with female indicated in blue and male in red. Higher composite VFQ-25 scores were associated with a better subjective vision-related QoL (Figure 4B).

## 4. Discussion

Assessing the impact of surgery on postoperative visual function in MSWM was the goal of this study. The VFQ-25 was used in this study because it is a well-established tool that has been proven reliable and valid in assessing VR-QoL in clinical studies in various conditions including pituitary adenoma, macular degeneration, stroke, and multiple sclerosis [10,27,28,29]. However, the present study represents the first to investigate the validated VFQ-25 in MSWM. According to the findings of the present study, patients’ postoperative visual performance deteriorates with increasing age at surgery and irregular tumor shape.

Numerous studies have recently explored the QoL of individuals with meningiomas. Nevertheless, most research has focused on overall QoL following surgery [8,17]. Different neuroanatomical tumor locations are associated with variations in postoperative visual function. A previous study demonstrated that the localization of the tumor has a significant impact on the postoperative visual outcome. Tumors situated at the tuberculum sellae exhibited the most favorable results, whereas those located at the diaphragma sellae or the medial sphenoid wing were more frequently associated with a postoperative decline in vision [30]. Another study about MSWM showed that tumor extension into the optic canal and its relationship to the internal carotid artery significantly influenced visual outcomes. Tumors involving these critical structures were associated with poorer postoperative visual prognosis [9].

Achieving a Simpson grade I resection in MSWMs is highly challenging and often infeasible due to several factors. The proximity to critical neurovascular structures, including the internal carotid artery, optic nerve, and cranial nerves within the cavernous sinus, significantly increases the risk of surgical morbidity associated with complete resection. These tumors frequently invade the cavernous sinus and adjacent bone, making total removal of all abnormal tissue extremely risky [2,6]. Due to the proximity to critical neurovascular structures and brain nerves of MSWMs, Simpson grade I resections are not determined as part the institution’s treatment policy because of the known increased risk of CSF leaks and cranial nerve deficits after Simpson grade I resections [5]. Another major challenge for neurosurgeons arises from the tumor’s irregular shape and the location in relation to adjacent critical neurovascular structures in MSWMs. Musigmann et al. showed that machine learning algorithms identified irregular tumor shape as being associated with a higher risk of subtotal resection and challenging neurosurgical prospects compared to a regular tumor shape [31]. Important neighboring neuroanatomical structures and intraoperative neurosurgical complications can also lead to long-term consequences. A previous study demonstrated that cranial nerve dysfunction and a shorter progression-free survival were linked to tumor shape irregularities in MSWM [13]. On the other hand, findings based on prior research suggest that tumors with irregular shape demonstrate more aggressive peritumoral edema and have a strong tendency to infiltrate, leading to a higher WHO grade, which may also result in damage to nearby structures [32,33].

The second finding supports the hypothesis that poor postoperative visual function outcomes are associated with a higher age at time of surgery.

The impact of age on postoperative visual outcomes is multifaceted, extending beyond the effects of surgical intervention. Higher age has long been recognized as a risk factor for poorer outcomes in neurosurgical patients, and this is particularly relevant for conditions involving the visual pathways [34,35]. Age-related physiological changes, such as reduced visual acuity, diminished contrast sensitivity, and increased prevalence of ocular conditions like cataracts, glaucoma, and macular degeneration, can independently contribute to worsening visual function [36,37]. These factors may compound the effects of surgical trauma or tumor-related damage, leading to a more pronounced decline in vision postoperatively. Moreover, age-associated neural and vascular changes, such as reduced plasticity of the visual pathways and decreased perfusion to the optic nerve and retina, may limit the potential for recovery in older patients. This adds to the evidence that age is not only a predictor of baseline visual function but also of the trajectory of recovery following surgery [38,39].

A recent study by Chen et al. compared short-term surgical outcomes and clinical characteristics between elderly and non-elderly patients with middle third parasagittal and parafalcine meningiomas. The study found that elderly patients had a higher incidence of preoperative comorbidities and postoperative complications, as well as longer hospital stays, compared to their younger counterparts. These findings underscore the importance of comprehensive preoperative assessment and tailored perioperative management in elderly patients to mitigate risks and improve surgical outcomes [40]. A recent case report by Lizana and colleagues described an exceptional instance of bilateral central retinal artery occlusion (CRAO) following resection of a frontal parasagittal meningioma. This rare postoperative complication resulted in profound bilateral vision loss and highlights the potential for vascular complications arising from intracranial tumor surgery. This case underlines the need for prompt recognition and intervention to mitigate irreversible visual damage. Although our study focuses on visual outcomes in SWMs, such findings underscore the broader spectrum of surgical risks that can significantly impair visual function [41].

May et al.’s study revealed that patients with skull base meningiomas who were older at the time of surgery had a higher incidence of postoperative neurological disorders [42]. In general, higher age is a well-known predictive factor for poorer functional outcome in meningioma patients [43,44,45,46]. Hence, there is also a potential confounding bias in elderly patients because of the natural course of visual aging. Nevertheless, our findings are supported by research in the field of ophthalmology, which shows that age is an independent factor for visual deterioration [47,48]. The current study did not show a correlation between cavernous sinus invasion and decline in visual function, in contrast to the findings of other studies [49,50]. It is important to consider that, in the present study, there were only a small number of cases with cavernous sinus invasion, and thus this study is underpowered to entirely answer questions surrounding MSWM with cavernous sinus invasion. In the present study, only seven patients received a diagnosis of cavernous sinus invasion.

## 5. Limitations

The present study’s retrospective design and the inclusion of patients with varied meningioma stages and Simpson resection grades introduce heterogeneity, which may influence the generalizability of the findings. However, this heterogeneity reflects the real-world diversity of medial sphenoid wing meningioma cases treated surgically. To address this, multivariable analyses were conducted to adjust for potential confounders, including age, tumor grade, extent of resection, and other clinical variables. Future studies could benefit from larger, more homogeneous cohorts or prospective subgroup analyses to validate these findings further.

## 6. Conclusions

This study evaluates an internationally recognized questionnaire to describe postoperative visual function in MSWM. The findings imply that patient-reported postoperative worsening of visual function is independently predicted by older age and irregular tumor shape. Notably, higher age at the time of surgery is a well-documented independent predictor of poorer neurosurgical outcomes, likely reflecting age-related vulnerability to neurological deficits and reduced capacity for recovery. These findings emphasize the importance of incorporating patient age and tumor shape into preoperative risk stratification and personalized surgical planning. The study’s outcomes provide actionable insights for improving patient counseling, optimizing perioperative management, and enhancing long-term QoL for MSWM patients.

## Figures and Tables

**Table 1:** Patient characteristics.

Characteristic	Value
Mean age at diagnosis	57.9 ± 12.9
Median time interval between surgery and interview, in months	108 ± 46.9
Sex	
Female	30 (71.4%)
Male	12 (28.6%)
WHO classification	
1	39 (92.9%)
2	3 (7.1%)
3	0 (0%)
Simpson resection grade	
I	0 (0%)
II	28 (66.6%)
III	2 (4.8%)
IV	12 (28.6%)
Cavernous sinus invasion	
Present	7 (16.7%)
Absent	35 (83.3%)
Adjuvant Radiotherapy	
Yes	2 (4.8%)
No	40 (95.2)
Neuroanatomical location	
Right side	20 (47.6%)
Left side	22 (52.4%)

## Data Availability

The original contributions presented in the study are included in the article, further inquiries can be directed to the corresponding authors.

## References

[1] Masalha W., Heiland D.H., Steiert C., Krüger M.T., Schnell D., Heiland P., Bissolo M., Grosu A.-L., Schnell O., Beck J. (2022). Management of Medial Sphenoid Wing Meningioma Involving the Cavernous Sinus: A Single-Center Series of 105 Cases. Cancers.

[2] Nakamura M., Roser F., Jacobs C., Vorkapic P., Samii M. (2006). Medial Sphenoid Wing Meningiomas: Clinical Outcome and Recurrence Rate. Neurosurgery.

[3] Goldbrunner R., Stavrinou P., Jenkinson M.D., Sahm F., Mawrin C., Weber D.C., Preusser M., Minniti G., Lund-Johansen M., Lefranc F. (2021). EANO Guideline on the Diagnosis and Management of Meningiomas. Neuro-Oncology.

[4] Wang J.Z., Patil V., Landry A.P., Gui C., Ajisebutu A., Liu J., Saarela O., Pugh S.L., Won M., Patel Z. (2024). Molecular Classification to Refine Surgical and Radiotherapeutic Decision-Making in Meningioma. Nat. Med..

[5] Schneider M., Schuss P., Güresir Á., Wach J., Hamed M., Vatter H., Güresir E. (2019). Cranial Nerve Outcomes After Surgery for Frontal Skull Base Meningiomas: The Eternal Quest of the Maximum-Safe Resection with the Lowest Morbidity. World Neurosurg..

[6] Meybodi A.T., Mignucci-Jiménez G., Lawton M.T., Liu J.K., Preul M.C., Sun H. (2023). Comprehensive Microsurgical Anatomy of the Middle Cranial Fossa: Part I—Osseous and Meningeal Anatomy. Front. Surg..

[7] San A., Rahman R.K., Sanmugananthan P., Dubé M.D., Panico N., Ariwodo O., Shah V., D’Amico R.S. (2023). Health-Related Quality of Life Outcomes in Meningioma Patients Based upon Tumor Location and Treatment Modality: A Systematic Review and Meta-Analysis. Cancers.

[8] Frances S.M., Murray L., Nicklin E., Velikova G., Boele F. (2024). Long-Term Health-Related Quality of Life in Meningioma Survivors: A Mixed-Methods Systematic Review. Neuro-Oncol. Adv..

[9] Chaichana K.L., Jackson C., Patel A., Miller N.R., Subramanian P., Lim M., Gallia G., Olivi A., Weingart J., Brem H. (2012). Predictors of Visual Outcome Following Surgical Resection of Medial Sphenoid Wing Meningiomas. J. Neurol. Surg. B Skull Base.

[10] Okamoto Y., Okamoto F., Hiraoka T., Yamada S., Oshika T. (2008). Vision-Related Quality of Life in Patients with Pituitary Adenoma. Am. J. Ophthalmol..

[11] Sanghani P., Beng Ti A., Kon Kam King N., Ren H. (2019). Evaluation of Tumor Shape Features for Overall Survival Prognosis in Glioblastoma Multiforme Patients. Surg. Oncol..

[12] Popadic B., Scheichel F., Pinggera D., Weber M., Ungersboeck K., Kitzwoegerer M., Roetzer T., Oberndorfer S., Sherif C., Freyschlag C.F. (2021). The Meningioma Surface Factor: A Novel Approach to Quantify Shape Irregularity on Preoperative Imaging and Its Correlation with WHO Grade. J. Neurosurg..

[13] Wach J., Naegeli J., Vychopen M., Seidel C., Barrantes-Freer A., Grunert R., Güresir E., Arlt F. (2023). Impact of Shape Irregularity in Medial Sphenoid Wing Meningiomas on Postoperative Cranial Nerve Functioning, Proliferation, and Progression-Free Survival. Cancers.

[14] Wadell H. (1935). Volume, Shape, and Roundness of Quartz Particles. J. Geol..

[15] Qiao N., Yang X., Li C., Ma G., Kang J., Liu C., Cao L., Zhang Y., Gui S. (2021). The Predictive Value of Intraoperative Visual Evoked Potential for Visual Outcome After Extended Endoscopic Endonasal Surgery for Adult Craniopharyngioma. J. Neurosurg..

[16] Mattogno P.P., D’Alessandris Q.G., Rigante M., Granata G., Di Domenico M., Perotti V., Montano N., Giordano M., Chiloiro S., Doglietto F. (2023). Reliability of Intraoperative Visual Evoked Potentials (iVEPs) in Monitoring Visual Function During Endoscopic Transsphenoidal Surgery. Acta Neurochir..

[17] Haider S., Taphoorn M.J.B., Drummond K.J., Walbert T. (2021). Health-Related Quality of Life in Meningioma. Neuro-Oncol. Adv..

[18] Fisher F.L., Zamanipoor Najafabadi A.H., van der Meer P.B., Boele F.W., Peerdeman S.M., Peul W.C., Taphoorn M.J.B., Dirven L., van Furth W.R. (2021). Long-Term Health-Related Quality of Life and Neurocognitive Functioning After Treatment in Skull Base Meningioma Patients. J. Neurosurg..

[19] Zamanipoor Najafabadi A.H., Genders S.W., van Furth W.R. (2021). Visual Outcomes Endorse Surgery of Patients with Spheno-Orbital Meningioma with Minimal Visual Impairment or Hyperostosis. Acta Neurochir..

[20] Louis D.N., Perry A., Reifenberger G., von Deimling A., Figarella-Branger D., Cavenee W.K., Ohgaki H., Wiestler O.D., Kleihues P., Ellison D.W. (2016). The 2016 World Health Organization Classification of Tumors of the Central Nervous System: A Summary. Acta Neuropathol..

[21] Nickels S., Schuster A.K., Singer S., Wild P.S., Laubert-Reh D., Schulz A., Finger R.P., Michal M., Beutel M.E., Münzel T. (2017). The National Eye Institute 25-Item Visual Function Questionnaire (NEI VFQ-25)—Reference Data from the German Population-Based Gutenberg Health Study (GHS). Health Qual. Life Outcomes.

[22] Goldstein J.E., Bradley C., Gross A.L., Jackson M., Bressler N., Massof R.W. (2022). The NEI VFQ-25C: Calibrating Items in the National Eye Institute Visual Function Questionnaire-25 to Enable Comparison of Outcome Measures. Transl. Vis. Sci. Technol..

[23] Owen C.G., Rudnicka A.R., Smeeth L., Evans J.R., Wormald R.P.L., Fletcher A.E. (2006). Is the NEI-VFQ-25 a Useful Tool in Identifying Visual Impairment in an Elderly Population?. BMC Ophthalmol..

[24] Takakusagi Y., Oike T., Shirai K., Sato H., Kano K., Shima S., Tsuchida K., Mizoguchi N., Serizawa I., Yoshida D. (2021). Validation of the Reliability of Machine Translation for a Medical Article from Japanese to English Using DeepL Translator. Cureus.

[25] Mangione C.M. The National Eye Institute 25-Item VFQ-25. Scoring Algorithm, Version 2000. https://www.nei.nih.gov/sites/default/files/2019-06/manual_cm2000.pdf.

[26] Youden W.J. (1950). Index for Rating Diagnostic Tests. Cancer.

[27] Suñer I.J., Kokame G.T., Yu E., Ward J., Dolan C., Bressler N.M. (2009). Responsiveness of NEI VFQ-25 to Changes in Visual Acuity in Neovascular AMD: Validation Studies from Two Phase 3 Clinical Trials. Investig. Ophthalmol. Vis. Sci..

[28] Rowe F.J., Hepworth L.R., Conroy E.J., Rainford N.E.A., Bedson E., Drummond A., García-Fiñana M., Howard C., Pollock A., Shipman T. (2019). Visual Function Questionnaire as an Outcome Measure for Homonymous Hemianopia: Subscales and Supplementary Questions, Analysis from the VISION Trial. Eye.

[29] Ma S.-L., Shea J.A., Galetta S.L., Jacobs D.A., Markowitz C.E., Maguire M.G., Balcer L.J. (2002). Self-Reported Visual Dysfunction in Multiple Sclerosis: New Data from the VFQ-25 and Development of an MS-Specific Vision Questionnaire. Am. J. Ophthalmol..

[30] Andrews B.T., Wilson C.B. (1988). Suprasellar Meningiomas: The Effect of Tumor Location on Postoperative Visual Outcome. J. Neurosurg..

[31] Musigmann M., Akkurt B.H., Krähling H., Brokinkel B., Henssen D.J.H.A., Sartoretti T., Gala Nacul N., Stummer W., Heindel W., Mannil M. (2022). Assessing Preoperative Risk of STR in Skull Meningiomas Using MR Radiomics and Machine Learning. Sci. Rep..

[32] Hashiba T., Hashimoto N., Maruno M., Izumoto S., Suzuki T., Kagawa N., Yoshimine T. (2006). Scoring Radiologic Characteristics to Predict Proliferative Potential in Meningiomas. Brain Tumor Pathol..

[33] Liu Y., Chotai S., Chen M., Jin S., Qi S.-T., Pan J. (2015). Preoperative Radiologic Classification of Convexity Meningioma to Predict the Survival and Aggressive Meningioma Behavior. PLoS ONE.

[34] Hu X., Ma Y., Jiang X., Tang W., Xia Y., Song P. (2023). Neurosurgical Perioperative Management of Frail Elderly Patients. Biosci. Trends.

[35] Löfgren D., Valachis A., Olivecrona M. (2022). Older Meningioma Patients: A Retrospective Population-Based Study of Risk Factors for Morbidity and Mortality After Neurosurgery. Acta Neurochir..

[36] Al-Namaeh M. (2022). Common Causes of Visual Impairment in the Elderly. Med. Hypothesis Discov. Innov. Ophthalmol..

[37] Remillard E.T., Koon L.M., Mitzner T.L., Rogers W.A. (2024). Everyday Challenges for Individuals Aging with Vision Impairment: Technology Implications. Gerontologist.

[38] Watanabe C., Imaizumi T., Kawai H., Suda K., Honma Y., Ichihashi M., Ema M., Mizutani K.-I. (2020). Aging of the Vascular System and Neural Diseases. Front. Aging Neurosci..

[39] Coleman-Belin J., Harris A., Chen B., Zhou J., Ciulla T., Verticchio A., Antman G., Chang M., Siesky B. (2023). Aging Effects on Optic Nerve Neurodegeneration. Int. J. Mol. Sci..

[40] Chen Z., Lin T., Liu D., Zeng Y., Zhang X., Deng B., Guo D., Shi T., Lu M. (2023). Comparison of Short-Term Surgery Outcomes and Clinical Characteristics Between Elderly and Non-Elderly Patients with Middle Third Parasagittal and Parafalcine Meningiomas. Neuropsychiatr. Dis. Treat..

[41] Lizana J., Dulanto Reinoso C.M., Aliaga N., Marani W., Montemurro N. (2021). Bilateral Central Retinal Artery Occlusion: An Exceptional Complication After Frontal Parasagittal Meningioma Resection. Surg. Neurol. Int..

[42] May M., Sedlak V., Pecen L., Priban V., Buchvald P., Fiedler J., Vaverka M., Lipina R., Reguli S., Malik J. (2023). Role of Risk Factors, Scoring Systems, and Prognostic Models in Predicting the Functional Outcome in Meningioma Surgery: Multicentric Study of 552 Skull Base Meningiomas. Neurosurg. Rev..

[43] Splavski B., Hadzic E., Bagic I., Vrtaric V., Splavski B. (2017). Simple Tumor Localization Scale for Estimating Management Outcome of Intracranial Meningioma. World Neurosurg..

[44] Zeng L., Wang L., Ye F., Chen J., Lei T., Chen J. (2015). Clinical Characteristics of Patients with Asymptomatic Intracranial Meningiomas and Results of Their Surgical Management. Neurosurg. Rev..

[45] Champeaux-Depond C., Weller J., Froelich S., Resche-Rigon M. (2021). A Nationwide Population-Based Study on Overall Survival After Meningioma Surgery. Cancer Epidemiol..

[46] Bartek J., Sjåvik K., Förander P., Solheim O., Gulati S., Weber C., Ingebrigtsen T., Jakola A.S. (2015). Predictors of Severe Complications in Intracranial Meningioma Surgery: A Population-Based Multicenter Study. World Neurosurg..

[47] Swenor B.K., Lee M.J., Varadaraj V., Whitson H.E., Ramulu P.Y. (2020). Aging with Vision Loss: A Framework for Assessing the Impact of Visual Impairment on Older Adults. Gerontologist.

[48] Sabeti F., Lane J., Rohan E.M.F., Essex R.W., McKone E., Maddess T. (2021). Relationships Between Retinal Structure and Function and Vision-Related Quality of Life Measures in Advanced Age-Related Macular Degeneration. Graefes Arch. Clin. Exp. Ophthalmol..

[49] Corniola M.V., Roche P.-H., Bruneau M., Cavallo L.M., Daniel R.T., Messerer M., Froelich S., Gardner P.A., Gentili F., Kawase T. (2022). Management of Cavernous Sinus Meningiomas: Consensus Statement on Behalf of the EANS Skull Base Section. Brain Spine.

[50] Beer-Furlan A., Priddy B.H., Jamshidi A.O., Shaikhouni A., Prevedello L.M., Ditzel Filho L., Otto B.A., Carrau R.L., Prevedello D.M. (2020). Improving Function in Cavernous Sinus Meningiomas: A Modern Treatment Algorithm. Front. Neurol..

